# Glyphosate-Eating Fungi: Study on Fungal Saprotrophic Strains’ Ability to Tolerate and Utilise Glyphosate as a Nutritional Source and on the Ability of *Purpureocillium lilacinum* to Degrade It

**DOI:** 10.3390/microorganisms9112179

**Published:** 2021-10-20

**Authors:** Veronica Spinelli, Andrea Ceci, Chiara Dal Bosco, Alessandra Gentili, Anna Maria Persiani

**Affiliations:** 1 Department of Environmental Biology, Sapienza University of Rome, Piazzale Aldo Moro 5, 00185 Rome, Italy; annamaria.persiani@uniroma1.it; 2 Department of Chemistry, Sapienza University of Rome, Piazzale Aldo Moro 5, 00185 Rome, Italy; chiara.dalbosco@uniroma1.it (C.D.B.); alessandra.gentili@uniroma1.it (A.G.)

**Keywords:** glyphosate, *Purpureocillium lilacinum*, saprotrophic fungi, bioremediation, Roundup, sarcosine, glyphosate biodegradation pathway, AMPA, tolerance index, biodegradation

## Abstract

Glyphosate is the most commonly used herbicide worldwide. Its improper use during recent decades has resulted in glyphosate contamination of soils and waters. Fungal bioremediation is an environmentally friendly, cost effective, and feasible solution to glyphosate contamination in soils. In this study, several saprotrophic fungi isolated from agricultural environments were screened for their ability to tolerate and utilise Roundup in different cultural conditions as a nutritional source. *Purpureocillium lilacinum* was further screened to evaluate the ability to break down and utilise glyphosate as a P source in a liquid medium. The dose–response effect for Roundup, and the difference in toxicity between pure glyphosate and Roundup were also studied. This study reports the ability of several strains to tolerate 1 mM and 10 mM Roundup and to utilise it as nutritional source. *P. lilacinum* was reported for the first time for its ability to degrade glyphosate to a considerable extent (80%) and to utilise it as a P source, without showing dose-dependent negative effects on growth. Pure glyphosate was found to be more toxic than Roundup for *P. lilacinum*. Our results showed that pure glyphosate toxicity can be only partially addressed by the pH decrease determined in the culture medium. In conclusion, our study emphasises the noteworthy potential of *P. lilacinum* in glyphosate degradation.

## 1. Introduction

Since the beginning of the Green Revolution, agriculture heavily relied on agrochemicals such as herbicides, pesticides, and chemical fertilisers to support high levels of food production to meet the demand [1]. However, due to inappropriate and excessive use in the past decades, agrochemicals were determined to have severe detrimental effects on the environment, such as eutrophication, ecosystem simplification, loss of ecosystem services, and loss of biodiversity and of soil quality [1,2]. Furthermore, the presence of agrochemical residues not only in the environment but also in processed food have raised concerns for their toxic effects on non-target organisms, including humans [3,4,5]. These concerns led also to the enactment of legislation requiring the limitation of agrochemical use (Directive 335 2009/128/EC and Regulation (EC) No 1107/2009 of the European Parliament and of the Council).

This recently developed awareness concerns in particular glyphosate, also known as N-(phosphonomethyl)-glycine. Being the active substance in more than 700 available commercial products [6,7,8], it is one of the most applied agrochemicals and undoubtedly the most commonly used herbicide worldwide, with an expected use of 740 to 920 thousand tons by 2025 [9,10,11,12]. Glyphosate as a post-emergent, non-selective systemic herbicide eliminating several weed species at the early growth stage is mainly used in agricultural land. Nevertheless, despite agricultural use accounting for 90% of the total usage, glyphosate is also utilised in non-agricultural land such as ruderal, industrial, or urban areas [10,13,14,15,16]. Its action is based on the inhibition of the 5-enolpyruvyl-shikimate-3-phosphate synthase, an essential enzyme responsible for the synthesis of amino acids in the shikimate pathway in plants and in some microorganisms. More precisely, glyphosate inhibits the production of the essential aromatic amino acids phenylalanine, tyrosine, and tryptophan and, consequently, the production of proteins and secondary metabolites [15,16]. In this way, glyphosate can generally kill treated plants in a few weeks and provide several other agricultural benefits, including tillage reduction and better crop production [13,15,17].

Although its toxicity to humans is still under debate, glyphosate residues in humans and animals have been detected and negative effects on animal metabolism, including oxidative stress, have been reported [10,18]. Residues have been detected also in crop plants and manure fertilisers, and negative effects have been observed on soil microorganisms, and microbes associated with plants and animals [16,19]. Among the negative effects due to glyphosate persistence in soil, an increase in bacterial and fungal drug resistance has also been reported. In fact, several microorganisms resistant to glyphosate were found to be resistant also to antibiotics with structural similarities to the herbicide [20,21,22]. Furthermore, glyphosate seems to be linked to plant pathogen increase. In fact, glyphosate causing a reduction in soil biodiversity also alters the key role fulfilled by the latter in avoiding the spread of plant pathogens in the environment. Moreover, a reduction in the resistance to pathogen infections has been observed in plants exposed even to very low concentrations of glyphosate due to residues in soil or water, hence favouring the increase in pathogen spread [10,21].

Despite a great number of commercial formulations being available, relatively little is known on the different toxicity of these compared with pure glyphosate. In this regard, for aquatic animals and mammalian cell lines, glyphosate has been consistently observed to be less toxic than Roundup [23]. However, Pochron et al. [8] reported that glyphosate and not Roundup is toxic to earthworms. The very limited data available suggest that commercial formulations are more toxic than glyphosate for fungi [24].

Due to the widespread use of glyphosate and its environmental persistence, public concerns about the contamination of soils, and surface and underground waters are increasing. For this reason, some countries have started to impose restrictions on its use. In fact, an analysis showed that a globally pervasive low contamination occurs in nearly all croplands where glyphosate is used [12]. Furthermore, glyphosate has been reported as a persistent contaminant, at relatively low values, in about 30% of global croplands. Additionally, aminomethylphosphonic acid (AMPA), one of glyphosate’s main metabolites, was found to be persistent in about 93% of croplands [12]. However, in the case of glyphosate ban, possible alternatives are still limited [13]. In 2017, the European Commission relicensed glyphosate for five additional years, and in the same year, 46.5 thousand tons of glyphosate active substances were sold in Europe [25,26].

As the world population is expected to grow, reaching nearly 9.7 billion by 2050, requiring an increase by up to 60% in food production, it is clear that a change towards a more sustainable agriculture is necessary [1,27]. Moreover, meeting the food demand will represent an even harder challenge since more than 33% of soils are already degraded worldwide [28]. In fact, human activities are overloading the global soil ecosystems as never before, impairing their functionality and stability and causing unreversible degradation and loss.

This need for a change has also been affirmed in the UN 2030 Agenda Sustainable Development Goals (SDG) that put the focus on the necessity to protect and restore this non-renewable resource to face the future global challenges. In fact, in several goals (SDG 2, 3, and 15), the importance of promoting sustainable agriculture that progressively improve land and soil quality and of protecting ecosystems and soil biodiversity that can avoid, reduce, and reverse land degradation is reaffirmed [29]. Among the threats for soil stability and functioning, contamination is one of the most important [3,4].

In this context, considering that, in soil [10,23], glyphosate can be slowly degraded by microorganisms, bioremediation can provide an environmentally friendly, cost effective, and feasible solution to glyphosate contamination in soils. In particular, fungal bioremediation or mycoremediation employs fungal species as multifunctional microorganisms, perfectly adapted to soil microhabitats [30,31], that can tolerate extreme environmental conditions [32,33,34]. Thanks to their functional traits, and highly potent and relatively non-specific enzymes, e.g., laccases and oxidoreductases, fungi can transform natural recalcitrant compounds as well as organic pollutants [34,35]. In general, co-metabolism of pesticides and other organic persistent pollutants is common in fungi that can transform xenobiotics into less toxic forms. In addition, some pollutants can be completely degraded by fungi, serving as sources of carbon and energy [34].

Several fungal strains, mainly belonging to few genera such as *Aspergillus*, *Trichoderma*, *Penicillium*, *Mucor*, and *Fusarium*, have already been reported to tolerate and/or degrade glyphosate. Known strains that tolerate glyphosate as pure molecule include *Penicillium chrysogenum*, which was able to grow in the presence of high concentrations, and *Fusarium solani* and *Fusarium oxysporum*, which showed tolerance to high doses of glyphosate [36,37]. Moreover, these strains were able not only to degrade it but also to utilise it as C or P sources [37,38]. Several studies, instead, focused on testing one or more glyphosate-based commercial formulations (e.g., Roundup ControlMax^®^, Roundup^®^ WG, Tornado plus, etc.), demonstrating that, among others, strains belonging to *Trichoderma* genus, such as *T. viride* and *T. inhamatum*, and to *Aspergillus* genus, such as *A. flavus*, *A. niger*, and *A. oryzae*, were able to tolerate and degrade these products [39,40,41,42]. Although in several studies the pure molecule has been compared with one or more commercial formulations, still, little is known about the different toxicities on fungi among them. For instance, Morjan et al. [24] tested four entomopathogenic fungi in the presence of seven glyphosate formulations, observing that several commercial formulations inhibited more fungal growth thanglyphosate.

Despite there being vast literature on fungi and glyphosate, still very limited are studies in this field. For instance, Arfarita et al. [41] reported that *T. viride* has been able to degrade glyphosate both in vitro and in field conditions; similarly, Kunanbayev et al. [42] showed that *T. inhamatum* has been successful also in the field. Furthermore, in a study on soil mycoflora, Sailaja and Satyaprasad [43] observed that there was a predominance of aspergilli, fusaria, penicillia, and *Trichoderma* species and that some *Trichoderma* spp. populations increased in the presence of glyphosate.

Currently, two main glyphosate microbial degradation pathways are known. One involves the C–N bond cleavage leading to the production of AMPA further metabolised to methylamine, and the other involves the C–P bond cleavage releasing sarcosine, which can be processed into glycine and formaldehyde [10,16].

Some fungal species have been reported to transform glyphosate into AMPA and to utilise it as the sole P source [10,15,16]. For instance, in *Aspergillus oryzae* A-F02, AMPA is further metabolised to methylamine, which is then metabolized into other products, suggesting that glyphosate could be used also as N or C sources [44]. In addition, Correa et al. [45] observed that the *Penicillium* 4A21 strain produced both AMPA and sarcosine as glyphosate degradation metabolites. Similarly, Adelowo et al. [46] detected AMPA and sarcosine working on *Trichoderma viridae*, *Aspergillus niger*, and *Fusarium oxysporum*. However, the glyphosate degradation mechanisms in fungi are still widely unknown and other metabolic pathways could be discovered.

In this study, several saprotrophic fungi were tested in the presence of glyphosate commercial formulation in different cultural conditions to select fungal strains able to tolerate and utilise glyphosate as a nutritional source. In this way, new strains representing useful bioresources can enlarge the known pool of candidates suitable for developing a feasible and sustainable strategy for glyphosate bioremediation.

Therefore, this study aimed to evaluate (1) fungal tolerance to glyphosate commercial formulation; (2) the fungal ability to use glyphosate as C or P sources; (3) the dose–response effect to Roundup exposure; (4) the ability of one selected fungal species, *Purpureocillium lilacinum*, to break down and utilise glyphosate as a P source in a liquid medium; and (5) the difference in toxicity between pure glyphosate and commercial formulation for *P. lilacinum*.

## 2. Materials and Methods

### 2.1. Screening of Fungal Strains for Glyphosate Commercial Formulation Tolerance

Eighteen fungal strains, previously isolated from agricultural environments and currently preserved at the culture collection of the Fungal Biodiversity Laboratory (FBL) (Sapienza, University of Rome), were screened to assess their tolerance to Roundup, one of the most common glyphosate commercial formulations. Prior to the experiment the strains have been reactivated and maintained at 25 °C in the dark on Malt Extract Agar (MEA) prepared according to the following composition (g/L in distilled water): malt extract, 20; peptone, 1; dextrose, 20; and bacto agar, 20. All components were purchased from Becton Dickinson (Sparks, MD, USA). Taking into account that, in the field, fungi are exposed to commercial formulations of glyphosate, tolerance tests were performed using Roundup Power 2.0^®^ (Bayer CropScience Srl, Milan, Italy) (RU). This product is a widely utilised glyphosate-based herbicide, nominally containing 360 g/L of pure glyphosate acid as a potassium salt. To evaluate a possible dose-dependent response, tolerance to RU was assessed trough a plate screening at two final concentrations corresponding to 1 mM and 10 mM of glyphosate, based on the reported nominal content, in 25 mL of Potato Dextrose Agar (PDA). The PDA was prepared according to the following composition (g/L in distilled water): potato dextrose broth, 24, and bacto agar, 20. Both components were purchased from Becton Dickinson (Sparks, MD, USA). Two RU solutions were prepared and filter-sterilised using 0.2 μm Whatman sterile syringe filters. An appropriate amount of RU solution was added in each plate contextually with molten PDA and homogenised before solidification. Control plates without RU were also prepared. One 6 mm diameter plug, taken from the actively growing margin of a 7-day old stock culture using a sterile cork borer, was inoculated in each plate. The assays were carried out in triplicate. The plates were incubated for 14 days in the dark at 25 °C, and diametric measurements of the fungal colonies were recorded daily. The growth responses to RU were evaluated through the R_t_:R_c_ (%) index defined as the ratio of the colony extension growth rates in the presence (R_t_) or absence (R_c_) of RU: R_t_:R_c_ (%) = (Growth rate of treated mycelium/Growth rate of control mycelium) × 100 [47].

### 2.2. Screening of Fungal Strains for Their Ability to Utilise Glyphosate as Nutritional Sources of C or P

Ten fungal species (Table 1) that were able to tolerate RU at 10 mM on PDA were further screened for their ability to utilise RU as nutritional sources of phosphorus (P) and carbon (C). Strains were tested on Czapeck dox agar (CDA) in enrichment conditions either for P (CDA P−) or C (CDA C−), according to the method previously applied in analogous studies [37,39]. CDA was prepared according to the following composition (g/L distilled water): sucrose, 30; NaNO_3_, 3; K_2_HPO_4_, 1; MgSO_4_•7H_2_O, 0.5; KCl, 0.5; FeSO_4_•7H_2_O, 0.01; and Bacto agar, 20. CDA P− was prepared following the abovementioned composition without K_2_HPO_4_, while CDA C− was prepared without sucrose. All chemicals were purchased from Merck (Darmstadt, Germany), except Bacto agar, which was purchased from Becton Dickinson (Sparks, MD, USA). To assess a potential dose-dependent response, the assays were set up at 1 mM and 10 mM of RU concentration. The controls were prepared on non-supplemented CDA, and the negative controls were prepared on CDA P− and CDA C− to assess the potential exploitation of agar and trace residues as P or C sources. Plate preparation and inoculation were the same as described in Section 2.1. The experiments were carried out for 14 days, in the dark at 25 °C. Growth responses and RU utilisation as a nutritional source were investigated by diametric measurements and tolerance index R_t_:R_c_ (%). The tolerance index was calculated utilising the growth rate control on CDA, which represents a growth baseline for the strains in a stress-free condition.

### 2.3. Screening of Purpureocillium lilacinum for Glyphosate and Roundup Breakdown and Utilisation as P Source in Liquid Culture Medium

Based on previous test results and interest in other applications in agricultural biotechnology [48,49,50,51], *Purpureocillium lilacinum* was selected for further screening. In order to evaluate the difference in toxicity between the pure molecule of glyphosate (GLY) and the commercial product (RU), the species was tested in a liquid Czapeck dox medium enrichment condition without phosphorus (CDB P−) at a concentration of 1 mM both GLY and RU. Moreover, to evaluate the influence of nutritional stress on the degradation process and possible co-metabolism, a treatment with RU concentration equivalent to 10 mM glyphosate active ingredient was set up in complete CDB and CDB P−. The two treatments in CDB P− at 1 mM and 10 mM were aimed also at evaluating a dose-dependent response. Furthermore, a negative control was set up in CDB P−. CDB was prepared according to the following composition (g/L distilled water): sucrose, 30; NaNO_3_, 3; K_2_HPO_4_, 1; MgSO_4_•7H_2_O, 0.5; KCl, 0.5; and FeSO_4_•7H_2_O, 0.01. CDB P− was prepared following the abovementioned composition without K_2_HPO_4_. The experiment was carried out in 100 mL Erlenmeyer flasks containing 50 mL of culture medium which were inoculated with a spore suspension of *P. lilacinum* at the final concentration of 1.55 × 10^7^ spores/mL. Prior to utilisation, all of the glassware was washed with 2 M HCl and rinsed multiple times with double distilled water (Millipore).

The experiments were carried out without shaking at 25 °C in the dark for four weeks. A sample of the culture media was recovered weekly, and biomass production was evaluated at the final time. Moreover, to evaluate the inhibition effect of GLYor RU on fungal growth, the inhibition index (%) was calculated: (dry weight of control mycelium − dry weight of treated mycelium)/dry weight of control mycelium × 100 [52]. The inhibition index was calculated using the CDB control that represents a growth baseline for the strains in a stress-free condition.

Measurements of medium pH were recorded weekly to evaluate the modifications during fungal growth.

### 2.4. Spectrophotometric Determination of P as a Glyphosate Breakdown Proxy

Unfortunately, due to a strong interference effect of the culture media, applying direct determination methods of glyphosate such as [53] was not possible. Therefore, the analysis of P released as a proxy of the degradation of glyphosate was carried out [37,46]. Orthophosphate was determined with a UV-Visible spectrophotometer (Shimadzu UV—1280) at 882 nm, using the ascorbic acid method [54]. Samples of the culture broth were filtered using a sterile syringe filter with a 0.45 μm pore size made of mixed cellulose esters (ClearLine^®^, Dominique Dutscher SAS, Brumath, France) and, when necessary, appropriately diluted before analysis. The P release due to glyphosate breakdown was evaluated as the difference between P detected in the inoculated sample and P in the chemical controls. However, in this kind of analysis, P determination is underestimated since the P uptaken during biomass growth is not detectable in the culture broth.

Analytical standards of glyphosate, AMPA, glycine and sarcosine were purchased from Sigma Aldrich/Merck (Milan, Italy). Formic acid (≥98% purity) and RS grade acetonitrile were purchased from the same supplier. Ultrapure water was produced through a Milli-Q Plus apparatus (Millipore, Bedford, MA, USA). The PVDF syringe filters (0.45 µm) were from Sigma-Aldrich.

Stock standard solutions were prepared at concentrations of 1 mg/mL by dissolving 1 mg of the compound (Ohaus DV215CD Discovery semi-micro and analytical balance, 81/210 g capacity, 0.01/0.1 mg readability) in ultrapure water in 1 mL volumetric flasks. Individual standard solutions at 1 and 10 ng/µL as well as a 2 ng/µL of multi-standard working solution were prepared by diluting the individual standard solutions in ultrapure water.

All of the samples (both the *P. lilacinum* inoculated Czapeck Dox Broth (CDB) media and the relative not inoculated blanks) were 10-fold water-diluted and filtered through 0.45 µm PVDF syringe filters before direct injection (5 µL) into the HPLC–MS system.

The chromatographic analysis was performed by means of a micro HPLC series 200 (PerkinElmer, Norwalk, CT, USA) equipped with a vacuum degasser, an autosampler, and a column oven. The target compounds were separated on a Kinetex F5 column (4.6 × 150 mm, 2.6 μm) from Phenomenex (Torrance, CA, USA) and kept at 298 K under isocratic conditions. The mobile phase was 90% water/10% acetonitrile, with both solvents added with 0.1% formic acid. A mixture of water and acetonitrile in the same proportion was used for washing the autosampler needle. The 1 mL/min flow rate was split by a post-column T-valve so that just 200 µL/min was entirely introduced into a 4000 Qtrap (AB SCIEX, Foster City, CA, USA) mass spectrometer equipped with an electrospray ionisation (ESI) probe on Turbo V source. The chromatograms were acquired in dual polarity (glyphosate and AMPA were detected in negative ion mode, sarcosine, and glycine in positive ion mode), with a needle current (NC) of 3 μA and a probe temperature of 723 K. As both the curtain (40 psi) and collision (4 mTorr) gas high-purity nitrogen were used, while air acted as the nebuliser (55 psi) and make-up (30 psi) gas. The chromatograms were acquired both in full scan (Q1) and in multiple reaction monitoring (MRM) mode by considering two MRM transitions for each analyte. Among these, the peak area of the most intense one (quantifier) was taken for the quantitative analysis. Calibration curves were obtained in solvent, since the matrix effect was minimised by the high dilution factor applied to the samples. The data were acquired and processed by Analyst^®^ 1.6.2 Software (AB Sciex, Foster City, CA, USA).

### 2.5. Screening of pH Medium Influence on Glyphosate and Roundup Breakdown by Purpureocillium lilacinum

Following the results of the abovementioned tests, a further experiment was necessary to determine whether the different biomass production between GLY and RU was either due to a different toxicity of the compounds or to the pH medium decrease caused by the addition of GLY. Therefore, the species was tested in CDB P− with and without tris(hydroxymethyl)aminomethane (TRIS) buffer [46,55] at a concentration of 1 mM GLY and RU. Furthermore, positive and negative controls were set up in CDB and CDB P− with and without TRIS. The experiment was carried out in 100 mL Erlenmeyer flasks containing 50 mL of culture medium which were inoculated with a spore suspension of *P. lilacinum* at the final concentration of 1.55 × 10^7^ spores/mL. The cultures were incubated without shaking at 25 °C in the dark for 2 weeks. The culture media and fungal biomass were recovered at the final time. The medium’s pH was measured to evaluate modifications due to the fungal growth. The culture broth was also analysed for the spectrophotometric determination of P as described in Section 2.4.

### 2.6. Statistical Analysis

All statistical analyses were carried out using the statistical software R (version 4.1.0) under the R-studio environment (version 1.4.1717). The data normality and homogeneity of variance were tested using the Shapiro–Wilk test (package stats), and Bartlett Test (package stats) or Levene Test (package lawstat) [56,57]. For normal homoscedastic data, a one-way analysis of variance (package stats) was performed, followed by the all-pairs comparison post hoc Tukey honest significant differences test (package stats). In some cases, the Welch test (package stats), followed by the all pairs comparison post hoc Dunnet’s T3 test (package PMCMRplus), or Friedman test (package muStat), followed by the all-pairs comparison post hoc Conover’s test (package PMCMRplus), were applied [58,59]. In Supplementary Material (Table S1), the statistical tests performed on the data of each screening are reported. A two-way ANOVA was performed to examine the effects of TRIS buffer addition and treatments on dry weight values (packages rstatix and emmeans) (Supplementary Material Figure S1) [60,61].

## 3. Results

### 3.1. Screening of Fungal Strains for Glyphosate Commercial Formulation Tolerance

The values of diametric growth in fungi incubated on PDA are shown in Table 2. All tested species were able to tolerate 1 mM RU, while only ten were able to tolerate 10 mM RU. *A. alliaceus* FBL 483 did not show the differences between the control and treatments at any tested concentrations. All of the other species in the 10 mM RU treatment showed significant growth reductions compared with control (*p <* 0.05). Only *A. flavipes* FBL 427 and *P. lilacinum* FBL 478 showed no differences between 1 mM RU and 10 mM RU but showed significant differences between control and 10mM RU. Most of calculated R_t_:R_c_ values for the 1 mM RU treatment showed values higher than 80%, indicating a strong tolerance of tested species (Table 3). R_t_:R_c_ values at 10 mM RU showed values higher than 80% only for *Mucor* sp. FBL 476 and *A. alliaceus* FBL 483.

### 3.2. Screening of Fungal Strains for Their Ability to Utilise Glyphosate as Nutritional Source of C or P

All tested species were able to grow in all treatments on CDA C− (Table 4 and Table 5). However, all the tested species were significantly affected by the presence of 10 mM RU in comparison with control (*p <* 0.05). *A. alliaceus* FBL 483, *A. flavipes* FBL 427, *Mucor* sp. FBL 476, *P. nitens* FBL 504, and *Trichoderma* sp. FBL 650 were not significantly affected by the presence of 1 mM RU in comparison with the control (*p <* 0.05). The growth of all tested species in the 10 mM RU treatment was significantly affected in comparison with 1 mM RU (*p <* 0.05). The calculated R_t_:R_c_ values for 1 mM RU were higher than 70% for all tested species with the only exception of *A. affinis* FBL 535 and *C. rosea* FBL 422, disclosing high tolerance.

All tested species were also able to grow in both RU treatments on CDA P− (Table 4 and Table 5). Although the diametric growth of all species was significantly reduced in the treatment with 10 mM RU in comparison with the control (*p <* 0.05), no significant differences in fungal growth in the treatment with 1 mM RU in comparison with the control were observed. With the only exception of *M. marquandii* FBL 484, all R_t_:R_c_ values for 1 mM RU were higher than 70%. The growth of all tested species in the 10 mM RU treatment was significantly reduced in comparison with the 1 mM RU treatment (*p <* 0.05). Tolerance indices for *A. affinis* FBL 535 and *A. alliaceus* FBL 483 at 1 mM RU were higher than 100%, suggesting a stimulation effect due to the presence of glyphosate.

**Figure 1:**
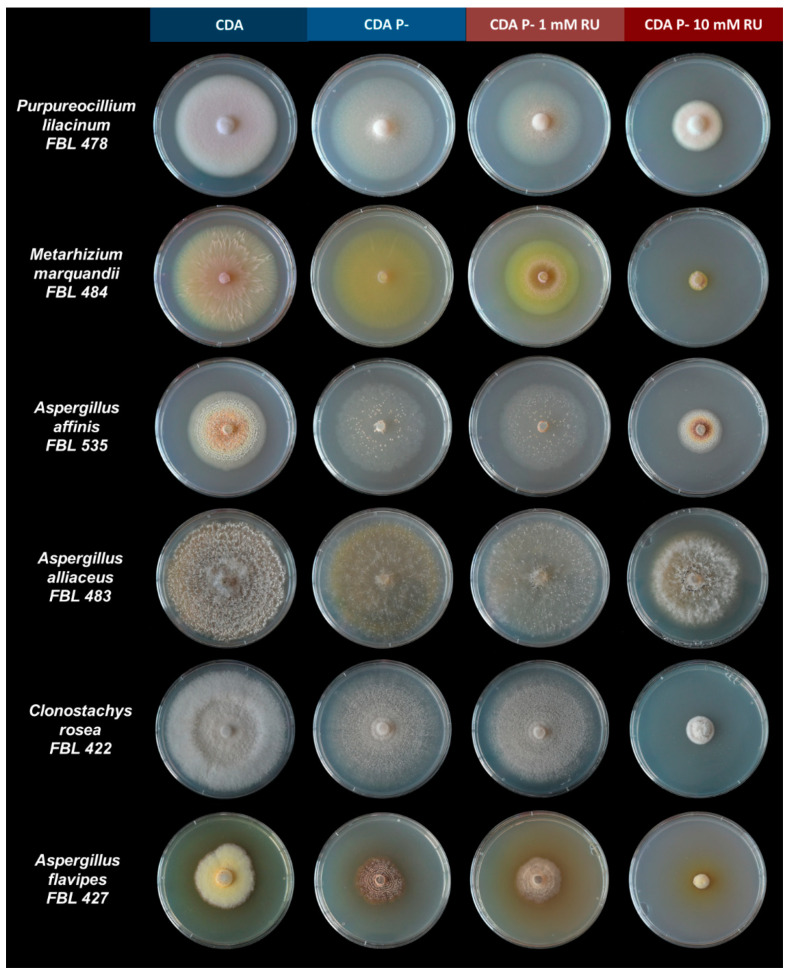
Morphological characteristics of the selected strain colonies in the controls on Czapek Dox Agar (CDA), negative controls on Czapek Dox Agar without phosphorus (CDA P−), 1 mM RU treatment on Czapek Dox Agar without phosphorus (CDA P− 1 mM RU), and 1 mM RU treatment on Czapek Dox Agar without phosphorus (CDA P− 10 mM RU) at 14 days of incubation.

In both the negative controls CDA C− and CDA P−, fungi were able to grow, probably exploiting agar as nutritional source (Table 4 and Table 5, Figure 1). However, it is worthwhile to note that the fungal mycelia were morphologically different from the control and, in most of cases, explorative (Figure 1).

### 3.3. Screening of Purpureocillium lilacinum for Glyphosate and Roundup Breakdown and Utilisation as a P Source in Liquid Culture Medium

#### 3.3.1. Evaluation of Fungal Growth through Biomass Production and Inhibition Index

The dry weight values of *P. lilacinum* FBL 478 after four weeks of growth and the inhibition index (%) values are shown in Table 6. The data showed that the presence of glyphosate or Roundup significantly reduced biomass production in comparison with the control (*p <* 0.05), inducing an inhibition of fungal growth ranging from 39.3% to 84.1%. While both RU and GLY caused relevant reductions in biomass production at 1 mM concentration, the dry weight in the latter was significantly lower (*p <* 0.001) and the lowest overall for all treatments (*p <* 0.05). By determining an 84.1% inhibition, GLY was way more toxic than RU, which caused a 58.2% inhibition. The dry weights of the CDB P− + 1 mM RU and CDB P− + 10 mM RU treatments were not significantly different; therefore, no dose-depending response in terms of fungal growth was observed for RU. However, CDB P− + 10 mM RU showed higher dry weight values and a lower inhibition index compared with CDB P− + 1 mM RU. Moreover, these treatments’ dry weights were higher than those of the negative control (*p <* 0.05), indicating the use of RU as a P source.

Furthermore, no difference between treatment CDB P− + 10 mM RU and CDB + 10 mM RU was observed; therefore, the difference in nutritional condition (complete medium+ RU/RU enrichment) did not affect the biomass production, showing very close inhibition percentages.

In general, all treatments in CDB P− showed lower biomass production than the control (CBD), and 1 mM GLY was even lower than negative control (CBD P−) (*p <* 0.05).

#### 3.3.2. Evaluation of the pH of Medium Modification during 4 Weeks of Incubation

The culture media pH values during the 4 weeks of incubation are shown in Table 7. All pH values of the chemical controls remained stable at their initial values during the four weeks.

In the first week, *P. lilacinum* was able to increase the pH of CDB− to 6.6 compared with its chemical control (5.0) (*p <* 0.05), while no variation was observed in the CBD pH. Instead, during the second week, *P. lilacinum* lowered the CDB pH value to 4.7 while increased it to 5.5 and 5.8 during the third and fourth weeks, respectively. In all of the other treatments, *P. lilacinum* increased the pH of the medium during the four weeks of growth. *P. lilacinum* in the CDB P− + 1 mM GLY treatment progressively increased the pH in comparison with its chemical control (3.1) up to pH 4.5 at the fourth week (*p* < 0.05); however, this treatment showed the lowest pH values in all 4 weeks compared with all the other treatments (*p <* 0.05). The low pH of this treatment’s medium was caused by glyphosate addition to a medium without phosphate or another buffering agent. The addition of Roundup also acidified the medium of the RU treatments, but the pH values were ≥ 4.5, probably due to the presence of additives with buffering action. In fact, the pH values of the CDB P− + 1 mM RU, CDB P− + 10 mM RU, and CDB + 10 mM RU chemical controls were, respectively, 4.6, 4.5, and 4.7 and were statistically different from their respective treatments inoculated with *P. lilacinum*. In both the treatments at 10 mM RU, *P. lilacinum* progressively increased the pH of the medium up to 5.8 and to 5.7, respectively, in the CDB P− + 10 mM RU and CDB + 10 mM RU treatments. It is interesting to note that, despite almost the same starting pH value, *P. lilacinum* in the CDB P− + 1 mM RU showed a statistically significantly higher increase in the medium’s pH compared with the CDB P− + 10 mM RU treatment, reaching a pH value of 7.1 by the fourth week.

At the fourth week, no differences in the medium’s pH between CDB− (pH = 7.1) and CDB− with 1 mM RU (pH = 7.2) and between CDB− with 10 mM RU (pH = 5.8) and CDB with 10 mM RU (pH = 5.7) were observed. In all of these cases, *P. lilacinum* increased the pH medium by about one unit in 4 weeks.

#### 3.3.3. Spectrophotometric Determination of Available P in the Culture Medium

The phosphorus concentrations in the culture media during the four weeks of incubation are reported in Table 7. Overall, in none of the biological treatments with *P. lilacinum* was an increase in P concentration observed. In the CDB P− and CDB P− + 1 mM GLY treatments’ chemical controls, no P concentration was detected, while negligible P concentrations were detected in the CDB P− + 1 mM RU and CDB P− + 10 mM RU treatments’ chemical controls (respectively, 0.2 and 0.8 mg/L), likely due to the P traces present in RU. The P concentrations in treatments’ chemical controls remained stable during the 4 weeks of incubation. *P. lilacinum* biological control CDB 478 samples showed lower P concentration (195 mg/L P) in comparison with the chemical control (204 mg/L P). An analogous P concentration reduction of about 10 mg/L was observed in CDB + 10 mM RU 478 (184 mg/L P) compared with the respective chemical control (194 mg/L P); however, in both cases, the differences were not statistically significant. Interestingly, the P concentration detected in these biological treatments compared with those of the chemical control showed a decrease by the first week, and later on, during the following three weeks, no further reduction was observed. No P was detected in the culture media of treatments CDB P−, CDB P− + 1 mM GLY and CDB P− + 1 mM RU inoculated with *P. lilacinum*.

CDB P− + 10 mM RU inoculated with *P. lilacinum*, despite showing very low P concentration, was the only treatment in which a progressive reduction in P concentration occurred during the four weeks of incubation.

#### 3.3.4. HPLC–MS Glyphosate Degradation Analysis of *P. lilacinum* Screening in Liquid Culture Medium

**Figure 2:**
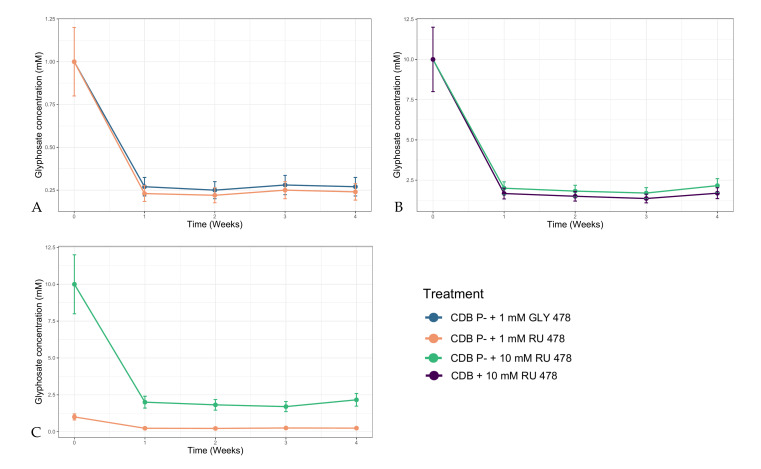
Glyphosate concentration (mM) during the four weeks of incubation: (**A**) glyphosate concentration trend in CDB P− + 1 mM GLY 478 and CDB P− + 1 mM RU 478, (**B**) glyphosate concentration trend in CDB P− + 10 mM RU 478 and CDB + 10 mM RU 478, and (**C**) glyphosate concentration trend in CDB P− + 10 mM RU 478 and CDB P− + 1 mM RU 478.

The quantitative analysis of CDB media with or without phosphorus, both at 10 mM in commercial glyphosate (Roundup), was conducted on different aliquots at time zero (not inoculated sample) and once a week after the inoculation of *P. lilacinum.* As represented in Figure 2, the degradation of glyphosate, in which total loss reached 80%, occurred within the first week from the cultural media inoculation and then remained constant until the fourth week. A similar trend was also obtained for the samples at a 1 mM GLY/RU concentration.

Identification of the possible metabolites (sarcosine, AMPA, glycine [44]) in real samples was carried out working in HPLC–Q1 full scan; however, it was particularly challenging due to their polarity, low masses, and low concentrations occurring in highly complex matrices. The extracted ion chromatogram at 90.1 m/z showed a chromatographic peak of low intensity, which matched the retention time of the standard solution of sarcosine; the latter was identified as the only metabolite in the samples starting from the third week after fugal inoculation.

### 3.4. Screening of the Influence of the Medium’s pH on Glyphosate and Roundup Breakdown by Purpureocillium lilacinum

#### 3.4.1. Evaluation of Fungal Growth in Buffered Media

The dry weight values of *P. lilacinum* FBL 478 after two weeks of growth are shown in Table 8. CDB + TRIS showed higher dry weights than CDB (*p <* 0.05). The addition of glyphosate or Roundup affected the fungal growth significantly, causing lower dry weights compared with the control (*p <* 0.05) and therefore a percentage of inhibition ranging from 31.6% to 77.2%. The dry weight in CDB P− + 1 mM GLY + TRIS was found to be significantly higher than in CDB P− + 1 mM GLY (*p <* 0.05), and inhibition in the treatment buffered with TRIS showed lower values (60.6%) than in the treatment without it (77.2%). Even though dry weights in CDB P− + 1 mM RU + TRIS showed slightly higher values than those in CDB P− + 1 mM RU, these two treatments were not statistically different. Moreover, 1 mM RU treatments did not show a statistically significant difference when compared with CDB P− + 1 mM GLY + TRIS. Finally, no statistically significant differences between CDB P− and CDB P− + TRIS as well as between these treatments and those with 1 mM GLY were observed. These findings were confirmed also by the two-way ANOVA, revealing a statistically significant interaction between the buffer addition and the treatment influencing dry weights in all treatment with the exception of P− + 1mM RU.

#### 3.4.2. Evaluation of the Modification of the Medium’s pH after Two Weeks of Incubation in Buffered Media

The pH values of the medium after two weeks of growth are shown in Table 8. CDB and CDB + TRIS pH after *P. lilacinum* growth resulted in lower values than their chemical controls (*p <* 0.05). In CDB P−, *P. lilacinum* caused a basification of the medium (*p <* 0.05), while in CDB P− + TRIS, an acidification (*p <* 0.05). It is interesting to note that considering two different starting pH, respectively 4.8 and 7.2, the final pH in both treatments was almost the same (6.7 and 6.8) showing no significant differences. In CDB P− + 1 mM GLY the fungal growth caused a significant decrease in the medium pH when compared with its respective chemical control. Instead, the pH of CDB P− + 1 mM GLY chemical control (3.1) resulted in significantly lower values than those of the respective biological treatment (3.8) (*p <* 0.05). This observed pH value was very close to 3.6, i.e., the pH observed at the second week of growth in the previous experiment (Table 7). All treatments buffered with TRIS, showed pH ≥ 6.8 in chemical controls, despite the addition of GLY or RU. A similar pattern to that observed for GLY was observed also for RU. In fact, in CDB P− + 1 mM RU *P. lilacinum* increased the pH of the medium, while in CDB P− + 1 mM RU+ TRIS, caused an acidification. No statistical difference between the pH of the biological treatments in CDB P− + 1 mM GLY and CDB P− + 1 mM RU was observed.

#### 3.4.3. Spectrophotometric Determination of Available P in Buffered Culture Media

Phosphorus concentration in culture media after two weeks of fungal growth are reported in Table 8. No P was detected in CDB P− and CDB P− + 1 mM RU + TRIS, while very low P concentrations, probably due to P traces in RU and TRIS, were detected in CDB P− + TRIS, CDB P− + 1 mM GLY, CDB P− + 1 mM GLY + TRIS and CDB P− + 1 mM RU chemical controls. In the abovementioned treatments inoculated with *P. lilacinum* no P was detected. *P. lilacinum* caused a reduction in P concentration in CDB from 194 mg/L in the control to 188 mg/L in the respective treatment and from 195 mg/L to 182 mg/L in CDB + TRIS.

## 4. Discussion

This study evaluated the in vitro ability of several fungal strains to tolerate and utilise Roundup, one of the most used glyphosate’s commercial formulation, as a nutritional source. All 18 tested strains were able to tolerate 1mM RU; however, only ten tolerated the 10 mM concentration, suggesting that, in the optimal culture condition, there is a dose-dependent response.

In this study’s test condition, none of the strains belonging to the Basidiomycota phylum (*P. ostreatus*, *T. hirsuta*, *T. versicolor*, *G. frondosa*, and *M. polyspora*) were able to tolerate RU at the highest concentration, while all except *M. polyspora* FBL 503 showed high tolerance at 1 mM concentration. In a previous study on glyphosate bioremediation, *P. ostreatus* was not able to degrade glyphosate and tolerated lower glyphosate concentrations compared with the highest one tested in this study [62]. Although there are few studies on basidiomycetes’ glyphosate tolerance, the bioremediation potential of ligninolytic enzymes they possess have been studied more thoroughly. Laccase from *Pleurotus* sp. were successfully tested with glyphosate [63] and laccases and other enzymes from *T. versicolor* were positively tested for glyphosate biodegradation [64]. *G. frondosa* and *T. hirsuta* are known to possess ligninolytic enzymes with a potential in the biodegradation of persistent organic pollutants, even though they have not been studied with glyphosate [65,66].

Most of the tested fungi belonging to the Ascomycota and Mucoromycota phyla were able to tolerate glyphosate at a 1 mM concentration, and in particular, *A. alliaceus* and *Mucor* sp. were the only species with high tolerance at 10 mM RU. Strains belonging to the *Mucor* genus have already been reported to be tolerant to glyphosate and use it as C and P sources [38], while *A. alliaceus* has not been previously studied for its tolerance to glyphosate. However, considering our results in addition to previous reports on its ability to degrade high concentrations of atrazine, it is possible to affirm that this strain shows high potential in the bioremediation of organic pollutants [67]. Interestingly, all three strains belonging to the *Chaetomium* genus, despite showing a good tolerance at 1 mM concentration, were not able to tolerate glyphosate at the highest concentration. Considering that, previously, *C. globosum* was tested up to about 4 mM RU with little effect on growth [67], probably, the inhibition occurs at higher concentrations.

The species that tolerated 10 mM RU when tested in enrichment conditions were able to grow without added P and C even at the 10 mM concentration, with the only exception of *A. flavipes* and *P. nitens*, which both did not grow in CDA C− 10 mM RU. However, in both C− and P− treatments, morphological modifications occurred in the treatments with RU compared with the control and among the two treatments. Other than a diametric growth reduction, morphological modifications were mainly observed in terms of sporulation, pigmentation, and mycelium density. Interestingly, most of the strains at 10 mM concentration, despite a diametric growth reduction, showed a morphological aspect more similar to the control than those of the 1 mM treatment, in terms of pigmentation and mycelium density. However, all of the strains were able to grow on CDA P− and CDA C−, probably exploiting agar as a nutritional source. This is consistent with several other studies on fungi where negative controls, even in water agar, showed a relevant diametric growth [37,38,39]. In our study, however, mycelia in negative controls were mostly thin; explorative; and especially in CDA C−, less pigmented, showing visible differences from the RU treatments. Hence, despite plate assay agar representing an interference, making it difficult to unequivocally evaluate glyphosate utilisation as a nutritional source, this assay may surely represent a first-line screening to select promising species for further tests.

*A. alliaceus*, *A. affinis*, *A. flavipes*, *A. ustus*, *C. rosea*, *M. marquandii*, *M. polyspora*, *G. frondosa*, *T. hirsuta*, *P. nitens*, and *P. lilacinum* are reported for the first time in this study for their ability to tolerate and utilise RU as a nutritional source.

Among the strains showing a good potential and not previously studied for glyphosate remediation, *P. lilacinum* was selected for further testing because of the wide interest in its biotechnological application. In fact, *P. lilacinum*, being an entomopathogenic species, has garnered wide interest for biocontrol application. Furthermore, it has been studied also for plant growth promoting activities; phytopathogen biocontrol; and bioremediation of potentially toxic elements such as As, Cd, Cr, and Pb [68,69,70,71]. Moreover, *P. lilacinum* has already been reported to be tolerant to several other herbicides with active ingredients such as Pendimethalin, Pethoxamid, Clomazone, Chlorotoluron, and Imazamox [72].

Our data confidently report the tolerance and ability of *P. lilacinum* to degrade glyphosate, exploiting it as a P nutritional source.

An HPLC–MS glyphosate degradation analysis showed this strain’s ability to degrade 80% of the initial concentration of glyphosate as pure molecule or commercial formulation within a week. No quantitative differences in the degraded glyphosate or in the produced metabolites were observed between RU and GLY despite the differences in biomass development being consistent. No dose-dependent negative effect was observed in terms of glyphosate degradation considering the 1 mM and 10 mM RU concentration treatments. In fact, both showed 80% degradation of glyphosate initial concentration, with the 10 mM RU treatment also showing a statistically significant higher biomass production even if it was lower than the control in CDB. Considering that no differences in biodegradation occurred between CDB P− + 10 mM RU and CDB + 10 mM RU, it is possible to deduce that an alternative source of P in the medium does not affect glyphosate degradation. In fact, in CDB, degradation occurred to the same extent as that in CDB P−, where RU represents the only P source. This finding also suggests that the enzymes probably responsible for the glyphosate degradation in *P. lilacinum* are not induced by P starvation as for other microorganisms [10,45,55].

Among the known glyphosate degradation metabolites, sarcosine was the only one detected in the samples starting from the third week. This finding may suggest that *P. lilacinum* degrades glyphosate through the pathway that involves a C–P bond cleavage, causing the release of a phosphate group and sarcosine, where the latter may be further degraded upon releasing glycine and formaldehyde [44]. In our samples, glycine has never been detected probably due to either an incomplete pathway or the uptake of sarcosine and/or released glycine by the biomass, since it can be utilised as nutritional sources [73,74]. Uptake in biomass may also explain the reason why, despite glyphosate degradation occurring mainly during the first week, sarcosine is not detected before the third week.

The findings of this study are consistent with the results obtained on other fungal species by Adelowo et al. [46] (*Trichoderma viridae*, *Aspergillus niger*, and *Fusarium oxysporum*) and Correa et al. [45] (*Penicillium* sp., *Aspergillus* sp., and *Trichoderma* sp.). However, Adelowo and Correa also detected AMPA, the first metabolite reported in another known glyphosate degradation pathway in fungi, in their samples [44,45,46]. The AMPA pathway involves the cleavage of the C–N bond of glyphosate releasing AMPA as first step of degradation, which can either be degraded to methylamine and phosphate or to phosphoformaldehyde and, later, to formaldehyde [10,44,73]. Therefore, considering their results, it is possible to hypothesise that the same strain may operate at the same time through different pathways.

Interestingly, in none of *P. lilacinum* samples AMPA was detected. Unfortunately, due to the impossibility to detect methylamine with the applied analytical technique, it is not possible to confidently affirm if glyphosate was not degraded to AMPA at all or, if otherwise, it has been completely degraded. Since AMPA has been reported for its toxicity [45], the absence of its residues in *P. lilacinum* glyphosate degradation products makes it a particularly suitable candidate for bioremediation applications.

Considering the great potential showed by this strain, additional experiments aimed at further evaluating and confirming the degradation pathway would be valuable. To be effective for this purpose, future experiments would include both analyses of the culture filtrate and of the fungal biomass, with much shorter intervals between sampling times during the first week.

Despite the biomass growth values in treatments being significantly higher than in the negative control, indicating that P was obtained from glyphosate, no increases in the available P in the culture media were observed. In fact, all treatments, including the control in CDB, showed a decrease in the concentration of available P in the culture media within the first week. An analogous trend of concentration reduction has also been observed by Adelowo et al. and Afarita et al. [46,75,76]. The lack of available P increase in the medium may be explained by fungal uptake of the P released from glyphosate. It is interesting to note that the biomass produced in CDB P− + 10 mM RU is the same as that produced in CDB + 10 mM RU and that the decrease of P amount in inoculated CDB and CDB + 10 mM RU compared with their respective chemical controls are analogous. Therefore, it is possible to deduce that the presence of glyphosate did not cause a reduction in P uptake in the fungus. Despite providing valuable information on the fungal growth dynamics and validating the starting absence of P in enrichment culture media, the phosphorus concentration in the culture media was not shown to be a reliable proxy of glyphosate degradation.

Finally, our data indicate that, for *P. lilacinum*, GLY is more toxic than RU. Despite the strain being able to show a reduction of 80% in the concentration of both compounds and to exploit them as nutritional source, RU caused a lower reduction in biomass production than GLY in the four-week experiment. In fact, biomass production was strongly affected in the presence of GLY, being lower than in the medium amended with 10 mM RU. Part of this strong biomass production inhibition in GLY may have been linked to the very low pH, determined by the addition of GLY to the medium. In fact, despite a tolerance range of pH 2–10, *P. lilacinum* has an optimal pH of 6.5 [77]. This may also explain the lower biomass production even compared with the negative control, where, despite growth being based on spores’ P reservoirs, the pH was 5.0 and the fungus was able to increase it to 6.6 within the first week. In the two-week experiment, biomass production in the buffered medium amended with GLY was significantly higher than that in the unbuffered medium. Nevertheless, it did not show any statistically significant difference compared with biomass production in the buffered and unbuffered media amended with RU, hence pointing out pH involvement in higher GLY toxicity. A significant increase in biomass production in the buffered medium amended with GLY compared with the unbuffered one was observed. Therefore, a higher GLY toxicity is surely related to low pH, but there may also be other drivers of toxicity contributing to the final effect. However, this pH-related effect may be due to the test condition in the liquid medium, considering that, in soil, the pH decrease due to the GLY occurrence is counteracted thanks to adsorption and buffering phenomena. Our finding on the higher toxicity of GLY than RU disagrees with what is reported from Morjan et al. [24], according to whom several RU formulations, but not GLY, induced growth inhibition in four entomopathogenic fungi (*Beauveria bassiana*, *Metarhizium anisopliae*, *Nomuraea rileyi*, and *Neozygites floridana*). However, these opposing results may be due to the differences in test conditions, since Morjan et al. tested in a solid medium with the disk diffusion method, which could have prevented the lowering of medium’s pH and therefore its relative effects [24].

## 5. Conclusions

This study reports several strains not previously studied for their ability to tolerate and utilise glyphosate as a nutritional source, contributing to widen the knowledge on fungal strains representing potential bioresources for glyphosate bioremediation.

*P. lilacinum* is reported for the first time for its ability to degrade glyphosate to a considerable extent (80%) and to utilise it as P source. The *P. lilacinum*’s degradation performance was not affected by the increase in RU concentration; in fact, the higher amount of RU did not cause negative effects and even promoted a higher biomass production. Neither did the presence of alternative P sources cause a reduction in glyphosate degradation differently from what is reported in the literature for other microorganisms. Finally, in this study, it was observed that GLY is more toxic than RU for *P. lilacinum*, even though its toxicity is mainly determined by the pH reduction in culture media.

As pointed out from this study’s results, *P. lilacinum* represents a great candidate for the development of biotechnological applications for glyphosate remediation to support sustainable soil restoration in agricultural lands.

## Figures and Tables

**Table 1:** Fungal strains selected for the study. ^1^ Fungal strains tested for Roundup (RU) tolerance; ^2^ fungal strains tested for their ability to use RU as nutritional sources of C or P.

Strain		Phylum	FBL Code
*Aspergillus affinis* Davolos, Persiani, Pietr., & Maggi	^1,2^	Ascomycota	535
*Aspergillus alliaceus* Thom & Church	^1,2^	Ascomycota	483
*Aspergillus flavipes* (Bainier & R. Sartory) Thom & Church	^1,2^	Ascomycota	427
*Aspergillus ustus* (Bainier) Thom & Church	^1,2^	Ascomycota	420
*Chaetomium globosum* Kunze	^1^	Ascomycota	205
*Chaetomium* sp.	^1^	Ascomycota	498
*Chaetomium* sp.	^1^	Ascomycota	499
*Clonostachys rosea* (Link) Schroers, Samuels, Seifert, & W. Gams	^1,2^	Ascomycota	422
*Grifola frondosa* (Dicks.) Gray	^1^	Basidiomycota	450
*Metarhizium marquandii* (Massee) Kepler, S.A. Rehner & Humber	^1,2^	Ascomycota	484
*Minimedusa polyspora* (Hotson) Weresub & P.M. LeClair	^1^	Basidiomycota	503
*Mucor* sp.	^1,2^	Mucoromycota	476
*Phycomyces nitens* (C. Agardh) Kunze	^1,2^	Mucoromycota	504
*Pleurotus ostreatus* (Jacq.) P. Kumm.	^1^	Basidiomycota	566
*Purpureocillium lilacinum* (Thom) Luangsa-ard, Houbraken, Hywel-Jones, & Samson	^1,2^	Ascomycota	478
*Trametes hirsuta* (Wulfen) Lloyd	^1^	Basidiomycota	564
*Trametes versicolor* (L.) Lloyd	^1^	Basidiomycota	565
*Trichoderma* sp.	^1,2^	Ascomycota	650

**Table 2:** Diametric values of fungal colonies after fourteen days of growth exposed to Roundup (RU) on Potato Dextrose Agar (PDA) at 25 °C. The data are expressed as the mean ± standard error of independent biological replicates ^a^.

Diameter (mm)			
Strain	PDA	PDA 1 mM RU	PDA 10 mM RU
*Aspergillus affinis* FBL 535	77.3 ± 4.3	68.5 ± 1.8	37.2 ± 2.9 *^▪^
*Aspergillus alliaceus* FBL 483	86.0 ± 0.0	86.0 ± 0.0	86.0 ± 0.0
*Aspergillus flavipes* FBL 427	40.0 ± 2.0	26.7 ± 2.3	16.5 ± 2.0 *
*Aspergillus ustus* FBL 420	77.3 ± 0.4	46.3 ± 3.7 *	8.0 ± 1.0 *^▪^
*Chaetomium globosum* FBL 205	51.7 ± 1.4	31.7 ± 1.2	6.0 ± 0.0 *^▪^
*Chaetomium* sp. FBL 498	86.0 ± 0.0	84.0 ± 2.0	6.0 ± 0.0 *^▪^
*Chaetomium* sp. FBL 499	79.0 ± 5.1	67.3 ± 6.2	6.0 ± 0.0 *^▪^
*Clonostachys rosea* FBL 422	85.3 ± 0.7	73.0 ± 0.6 *	23.0 ± 2.1 *^▪^
*Grifola frondosa* FBL 450	40.3 ± 2.3	34.5 ± 0.9	6.0 ± 0.0 *^▪^
*Metarhizium marquandii* FBL 484	58.5 ± 2.5	32.3 ± 1.3 *	12.2 ± 0.6 *^▪^
*Minimedusa polyspora* FBL 503	77.7 ± 5.4	28.3 ± 3.2 *	6.0 ± 0.0 *^▪^
*Mucor* sp. FBL 476	86.0 ± 0.0	86.0 ± 0.0	76.0 ± 1.4 *^▪^
*Phycomyces nitens* FBL 504	86.0 ± 0.0	86.0 ± 0.0	19.0 ± 4.2 *^▪^
*Pleurotus ostreatus* FBL 566	86.0 ± 0.0	81.3 ± 1.2	6.0 ± 0.0 *^▪^
*Purpureocillium lilacinum* FBL 478	72.5 ± 0.6	65.0 ± 2.1	35.3 ± 2.3 *
*Trametes hirsuta* FBL 564	86.0 ± 0.0	77.3 ± 1.5	6.0 ± 0.0 *^▪^
*Trametes versicolor* FBL 565	86.0 ± 0.0	82.8 ± 1.4	6.0 ± 0.0 *^▪^
*Trichoderma* sp. FBL 650	86.0 ± 0.0	86.0 ± 0.0	61.5 ± 2.0 *^▪^

^a^ Asterisks (*) denote a significant difference between the treatments and the control while squares (^▪^) denote a significant difference between the treatments (post hoc Conover test, *p* < 0.05).

**Table 3:** Tolerance index (R_t_:R_c_) of the tested species exposed to Roundup on PDA.

R_t_:R_c_ (%) Tolerance Index		
Strain	1 mM RU	10 mM RU
*Aspergillus affinis* FBL 535	87.6	43.7
*Aspergillus alliaceus* FBL 483	100.0	100.0
*Aspergillus flavipes* FBL 427	60.8	30.9
*Aspergillus ustus* FBL 420	56.6	2.8
*Chaetomium globosum* FBL 205	56.2	0.0
*Chaetomium* sp. FBL 498	97.5	0.0
*Chaetomium* sp. FBL 499	84.0	0.0
*Clonostachys rosea* FBL 422	84.5	21.4
*Grifola frondosa* FBL 450	83.0	0.0
*Metarhizium marquandii* FBL 484	50.2	11.7
*Minimedusa polyspora* FBL 503	31.2	0.0
*Mucor* sp. FBL 476	100.0	87.5
*Phycomyces nitens* FBL 504	100.0	16.3
*Pleurotus ostreatus* FBL 566	94.2	0.0
*Purpureocillium lilacinum* FBL 478	88.7	44.1
*Trametes hirsuta* FBL 564	89.2	0.0
*Trametes versicolor* FBL 565	96.0	0.0
*Trichoderma* sp. FBL 650	100.0	69.4

**Table 4:** Diametric values of fungal colonies after fourteen days of growth at 25 °C on Czapeck dox agar (CDA) in enrichment conditions either for P (CDA P−) or C (CDA C−). The data are expressed as the mean ± standard error of independent biological replicates ^a^.

Diameter (mm)							
Strain	CDA	CDA P−	CDA P− 1 mM RU	CDA P− 10 mM RU	CDA C−	CDA C− 1 mM RU	CDA C− 10 mM RU
*Aspergillus affinis* FBL 535	52.7 ± 0.3	62.7 ± 0.9	60.0 ± 0.0	33.2 ± 1.6 *^•^^▪^	35.8 ± 1.4 *	38.5 ± 3.8 *	21.3 ± 2.2 *^•^^▪^
*Aspergillus alliaceus* FBL 483	81.2 ± 0.2	80.3 ± 0.2	81.3 ± 0.3	61.8 ± 2.3 *^•^^▪^	77.0 ± 2.0	72.0 ± 1.0	52.3 ± 0.3 *^•^^▪^
*Aspergillus flavipes* FBL 427	39.7 ± 0.9	32.7 ± 0.3	32.0 ± 0.0	11.7 ± 0.7 *^•^^▪^	55.3 ± 2.9 *	34.7 ± 0.7^•^	6.0 ± 0.0 *^•^^▪^
*Aspergillus ustus* FBL 420	64.2 ± 1.6	46.5 ± 1.3 *	54.8 ± 1.7	12.8 ± 1.6 *^•^^▪^	77.7 ± 1.3 *	49.8 ± 0.4 *^•^	7.2 ± 1.2 *^•^^▪^
*Clonostachys rosea* FBL 422	83.0 ± 0.6	81.3 ± 0.9	78.2 ± 0.6	19.2 ± 0.6 *^•^^▪^	82.3 ± 0.9	38.0 ± 0.9 *^•^	9.5 ± 0.3 *^•^^▪^
*Metarhizium marquandii* FBL 484	71.3 ± 2.7	69.2 ± 1.1	49.5 ± 0.5 *^•^	13.3 ± 0.2 *^•^^▪^	64.7 ± 2.2	52.7 ± 2.9 *	12.0 ± 0.3 *^•^^▪^
*Mucor* sp. FBL 476	86.0 ± 0.0	86.0 ± 0.0	86.0 ± 0.0	28.2 ± 1.8 *^•^^▪^	86.0 ± 0.0	86.0 ± 0.0	12.7 ± 0.4 *^•^^▪^
*Phycomyces nitens* FBL 504	86.0 ± 0.0	86.0 ± 0.0	86.0 ± 0.0	7.3 ± 0.3 *^•^^▪^	86.0 ± 0.0 *	82.0 ± 2.6 ^•^	6.0 ± 0.0 *^•^^▪^
*Purpureocillium lilacinum* FBL 478	69.7 ± 0.3	70.0 ± 0.0	57.3 ± 0.7 *^•^	33.8 ± 0.4 *^•^^▪^	66.5 ± 0.0	54.3 ± 0.2 *	28.5 ± 0.6 *^•^^▪^
*Trichoderma* sp. FBL 650	86.0 ± 0.0	86.0 ± 0.0	86.0 ± 0.0	39.7 ± 0.3 *^•^^▪^	86.0 ± 0.0	86.0 ± 0.0	21.3 ± 0.7 *^•^^▪^

^a^ Asterisks (*) denote a significant difference between the negative control or RU treatments, and the control; dots (^•^) denote a significant difference between the RU treatments and the negative control; and squares (**^▪^**) denote a significant difference between 1 and 10 mM RU treatments in the same cultural medium (post hoc Conover test, *p* < 0.05).

**Table 5:** Tolerance index (R_t_:R_c_) of the tested species exposed to Roundup as the only source of P or C on enriched CDA.

	CDA P−		CDA C−	
Strain	1 mM RU	10 mM RU	1 mM RU	10 mM RU
*Aspergillus affinis* FBL 535	115.7	58.2	69.6	32.9
*Aspergillus alliaceus* FBL 483	100.2	74.3	87.8	61.6
*Aspergillus flavipes* FBL 427	77.2	16.8	96.1	0.0
*Aspergillus ustus* FBL 420	84.0	11.7	75.6	2.0
*Clonostachys rosea* FBL 422	94.3	17.2	41.6	4.5
*Metarhizium marquandii* FBL 484	66.6	11.2	71.4	9.2
*Mucor* sp. FBL 476	100.0	27.7	100.0	8.3
*Phycomyces nitens* FBL 504	100.0	1.7	95	0.0
*Purpureocillium lilacinum* FBL 478	80.6	43.7	75.9	35.3
*Trichoderma* sp. FBL 650	100.0	42.1	100.0	19.2

**Table 6:** Inhibition index and dry weights of fungal biomass after four weeks of growth at 25 °C in liquid Czapeck dox medium (CDB) enrichment condition without phosphorus (CDB P−) at a concentration of 1 mM both pure glyphosate (GLY) and RU. The data for dry weights (g) are expressed as the mean ± standard error of biological independent replicates.

Treatment	Dry Weight (g)	Inhibition Index (%)
CDB 478	0.0241 ± 0.0013 ^A^	−
CDB P− 478	0.0065 ± 0.0004 ^B^	−
CDB P− + 1 mM GLY 478	0.0038 ± 0.0004 ^C^	84.1
CDB P− + 1 mM RU 478	0.0101 ± 0.0002 ^D^	58.2
CDB P− + 10 mM RU 478	0.0142 ± 0.0016 ^E^	41.0
CDB + 10 mM RU 478	0.0146 ± 0.0007 ^E^	39.3

The same superscript letters denote no significant difference between values (post hoc Dunnet T3 test, *p >* 0.05).

**Table 7:** The pH values for the medium after fungal growth for 1, 2, 3, and 4 weeks at 25 °C and the P concentration in the medium. The data are expressed as the mean ± standard error of independent replicates ^a^.

**pH**				
**Treatment**	**7 Days**	**14 Days**	**21 Days**	**28 Days**
CDB 478	5.7 ± 0.0 ^A^	4.7 ± 0.0 ^A^	5.5 ± 0.1 ^A^	5.8 ± 0.0 ^A^
CDB P− 478	6.6 ± 0.1 ^B^	6.6 ± 0.0 ^B^	6.8 ± 0.1 ^B^	7.2 ± 0.0 ^B^
CDB P− + 1 mM GLY 478	3.4 ± 0.0 ^C^	3.6 ± 0.1 ^C^	4.1 ± 0.3 ^C^	4.5 ± 0.1 ^C^
CDB P− + 1 mM RU 478	6.0 ± 0.0 ^A^	6.4 ± 0.1 ^B^	6.9 ± 0.1 ^B^	7.1 ± 0.1 ^B^
CDB P− + 10 mM RU 478	4.9 ± 0.0 ^D^	5.1 ± 0.0 ^D^	5.6 ± 0.0 ^A^	5.8 ± 0.0 ^A^
CDB + 10 mM RU 478	4.9 ± 0.0 ^D^	5.1 ± 0.0 ^D^	5.5 ± 0.1 ^A^	5.7 ± 0.1 ^A^
CDB Control	5.7 ± 0.0 ^A^	5.7 ± 0.0 ^E^	5.7 ± 0.0 ^A^	5.7 ± 0.0 ^A^
CDB P− Control	5.0 ± 0.1 ^D^	5.0 ± 0.0 ^D^	5.1 ± 0.2 ^D^	4.9 ± 0.0 ^C^
CDB P− + 1 mM GLY Control	3.1 ± 0.0 ^E^	3.1 ± 0.0 ^F^	3.1 ± 0.0 ^E^	3.1 ± 0.0 ^D^
CDB P− + 1 mM RU Control	4.6 ± 0.0 ^FG^	4.5 ± 0.0 ^G^	4.6 ± 0.0 ^C^	4.5 ± 0.0 ^C^
CDB P− + 10 mM RU Control	4.5 ± 0.0 ^F^	4.5 ± 0.0 ^G^	4.5 ± 0.0 ^C^	4.5 ± 0.0 ^C^
CDB + 10 mM RU Control	4.7 ± 0.0 ^G^	4.7 ± 0.0 ^A^	4.7 ± 0.0 ^C^	4.6 ± 0.0 ^C^
**Liquid Medium P Content (mg/L)**				
**Treatment**	**7 Days**	**14 Days**	**21 Days**	**28 Days**
CDB 478	195.3 ± 1.7 ^A^	195.4 ± 4.2 ^A^	195.9 ± 2.8 ^A^	195.9 ± 6.4 ^A^
CDB P− 478	0.0 ± 0.0	0.0 ± 0.0	0.0 ± 0.0	0.0 ± 0.0
CDB P− + 1 mM GLY 478	0.0 ± 0.0	0.0 ± 0.0	0.0 ± 0.0	0.0 ± 0.0
CDB P− + 1 mM RU 478	0.0 ± 0.0	0.0 ± 0.0	0.0 ± 0.0	0.0 ± 0.0
CDB P− + 10 mM RU 478	0.5 ± 0.1 ^B^	0.4 ± 0.0 ^B^	0.2 ± 0.0 ^B^	0.0 ± 0.0
CDB + 10 mM RU 478	183.8 ± 3.3 ^A^	185.4 ± 2.5 ^A^	186.7 ± 4.2 ^A^	184.7 ± 4.1 ^A^
CDB Control	204.0 ± 8.3 ^A^	207.6 ± 5.4 ^A^	206.0 ± 8.8 ^A^	204.3 ± 2.0 ^A^
CDB P− Control	0.0 ± 0.0	0.0 ± 0.0	0.0 ± 0.0	0.0 ± 0.0
CDB P− + 1 mM GLY Control	0.0 ± 0.0	0.0 ± 0.0	0.0 ± 0.0	0.0 ± 0.0
CDB P− + 1 mM RU Control	0.2 ± 0.0 ^C^	0.2 ± 0.0 ^C^	0.2 ± 0.0 ^B^	0.2 ± 0.0 ^B^
CDB P− + 10 mM RU Control	0.8 ± 0.0 ^D^	0.8 ± 0.0 ^D^	0.9 ± 0.1 ^C^	0.8 ± 0.1 ^C^
CDB + 10 mM RU Control	194.0 ± 2.0 ^A^	198.0 ± 6.6 ^A^	194.3 ± 11.7 ^A^	194.6 ± 5.9 ^A^

^a^ The same superscript letters denote no significant difference between values within the same timepoint column (post hoc Dunnet T3 test or Conover test, *p >* 0.05).

**Table 8:** Values of dry weights, inhibition index, medium pH, and P concentration after fungal growth for 2 weeks at 25 °C in different cultural conditions with or without tris(hydroxymethyl)aminomethane (TRIS) buffer. The data are expressed as the mean ± standard error of independent replicates ^a^.

	Dry Weight (g)	Inhibition Index (%)	pH	mg/L P
CDB 478	0.0182 ± 0.0010 ^A^	−	4.8 ± 0.09 ^A^	187.9 ± 3.7 ^A^
CDB + TRIS 478	0.0238 ± 0.0009 ^B^	−	5.9 ± 0.00 ^B^	181.9 ± 3.7 ^A^
CDB P− 478	0.0075 ± 0.0017 ^CD^	−	6.7 ± 0.02 ^C^	0.0 ± 0.0
CDB P− + TRIS 478	0.0029 ± 0.0010 ^E^	−	6.8 ± 0.00 ^D^	0.0 ± 0.0
CDB P− + 1 mM GLY 478	0.0041 ± 0.0005 ^C^	77.2	3.7 ± 0.02 ^E^	0.0 ± 0.0
CDB P− + 1 mM GLY + TRIS 478	0.0094 ± 0.0006 ^DF^	60.6	6.5 ± 0.00 ^F^	0.0 ± 0.0
CDB P− + 1 mM RU 478	0.0124 ± 0.0006 ^F^	31.6	6.4 ± 0.14 ^F^	0.0 ± 0.0
CDB P− + 1 mM RU + TRIS 478	0.0134 ± 0.0007 ^F^	43.8	6.0 ± 0.00 ^G^	0.0 ± 0.0
CDB Control	−	−	5.6 ± 0.01 ^H^	193.9 ± 2.7 ^A^
CDB + TRIS Control	−	−	7.2 ± 0.00 ^I^	195.1 ± 3.9 ^A^
CDB P− Control	−	−	4.8 ± 0.01 ^A^	0.0 ± 0.0
CDB P− + TRIS Control	−	−	7.2 ± 0.00 ^I^	<LOD ^◊^
CDB P− + 1 mM GLY Control	−	−	3.1 ± 0.00 ^L^	0.2 ± 0.0 ^B^
CDB P− + 1 mM GLY + TRIS Control	−	−	6.8 ± 0.00 ^D^	0.2 ± 0.0 ^B^
CDB P− + 1 mM RU Control	−	−	4.5 ± 0.01 ^M^	<LOD ^◊^
CDB P− + 1 mM RU + TRIS Control	−	−	6.8 ± 0.00 ^D^	0.0 ± 0.0

^◊^ value below limits of detection (LOD). ^a^ The same superscript letters denote no significant difference between values within the same parameter column (post hoc Tukey, Conover or Dunnet T3 test, *p >* 0.05).

## Data Availability

The data presented in this study are available on request from the corresponding authors.

## Supplementary Materials

The following are available online at https://www.mdpi.com/article/10.3390/microorganisms9112179/s1, Figure S1: Boxplot of the Simple pairwise comparison output, Table S1: Report of the performed statistical tests on each screenings’ data.
