# Supplementary material for: Glyphosate-Eating Fungi: Study on Fungal Saprotrophic Strains’ Ability to Tolerate and Utilise Glyphosate as a Nutritional Source and on the Ability of Purpureocillium lilacinum to Degrade It
Source: Microorganisms. 2021 Oct 20;9(11):2179. doi: 10.3390/microorganisms9112179 (PMC8623091; doi:10.3390/microorganisms9112179)
Supplement: Supplementary file 1 [file microorganisms-09-02179-s001.zip › microorganisms-1347478-supplementary.pdf]

Supplementary Material S1

Table S1.1. In the table are reported the performed tests on each screenings' data.

Screening	Outcome variable	Performed tests
Screening of fungal strains for glyphosate commercial formulation tolerance	Diameter (mm)	Friedman test followed by post-hoc Conover's test
Screening of fungal Strains for their ability to utilise glyphosate as nutritional source of C or P	Diameter (mm)	Friedman test followed by post-hoc Conover's test
Screening of *Purpureocillium lilacinum* for Glyphosate and Roundup breakdown and utilisation as P source in liquid culture medium	Dry weights (g)	Welch test followed by post-hoc Dunnet's T3 test
	pH	Welch test followed by post-hoc Dunnet's T3 test
	P concentration (mg/L)	Welch test followed by post-hoc Dunnet's T3 test
Screening of pH medium influence on Glyphosate and Round Up breakdown by *Purpureocillium lilacinum*	Dry weights (g)	One-way ANOVA followed by post-hoc Tukey honest significant differences test<br><br>Two-way ANOVA followed by Simple main effect and Simple pairwise comparisons
	pH	Friedman test followed by post-hoc Conover's test
	P concentration (mg/L)	Welch test followed by post-hoc Dunnet's T3 test

Two-way ANOVA was conducted to examine the Treatments and TRIS buffer addition effects on Biomass production (dry weights) in the Screening of pH medium influence on Glyphosate and Round Up breakdown by *Purpureocillium lilacinum*

The two-way ANOVA assumptions were verified through appropriate tests whose results were:

- Absence of extreme outliers;
- normally distributed residuals (Shapiro-Wilk's normality test $p > 0.05$); Normality assumption was also confirmed for each cell of design ($p > 0.05$);
- homogeneity of variance ($p > 0.05$).

A two-way ANOVA was conducted to examine the Treatments and TRIS buffer addition effects on Biomass production (dry weights). ANOVA showed a statistically significant interaction between the buffer addition and the treatment influencing dry weights, $F(3, 17) = 12.52$, $p = 0.00014$, eta2[g] = 0.69.

ANOVA Table

	Effect	DFn	DFd	F	p	p<.05	ges
1	Treatment	3	17	114.950	1.73e-11	*	0.953
2	Buffer	1	17	8.326	1.00e-02	*	0.329
3	Treatment:Buffer	3	17	12.515	1.45e-04	*	0.688

Consequently, an analysis of simple main effects has been performed with statistical significance adjusted with the Bonferroni method. Finally, a simple pairwise comparison analysis was performed. A statistically significant difference in dry weight means between TRIS presence and TRIS absence conditions was observed in all treatments with the exception of CDB P- 1mM RU ($p > 0.05$).

Table of Simple pairwise comparison

A tibble: 4 x 10

	Treatment	term	.y.	group1	group2	df	statistic	p	p.adj	p.adj.signif
*	<chr>	<chr>	<chr>	<chr>	<chr>	<dbl>	<dbl>	<dbl>	<dbl>	<chr>
1	CDB	Buffer	dw	no TRIS	with TRIS	17	-4.42	0.000371	0.000371	***
2	CDB P-	Buffer	dw	no TRIS	with TRIS	17	3.36	0.00376	0.00376	**
3	CDB P-+1 mM GLY	Buffer	dw	no TRIS	with TRIS	17	-3.82	0.00138	0.00138	**
4	CDB P-+1 mM RU	Buffer	dw	no TRIS	with TRIS	17	-0.681	0.505	0.505	ns

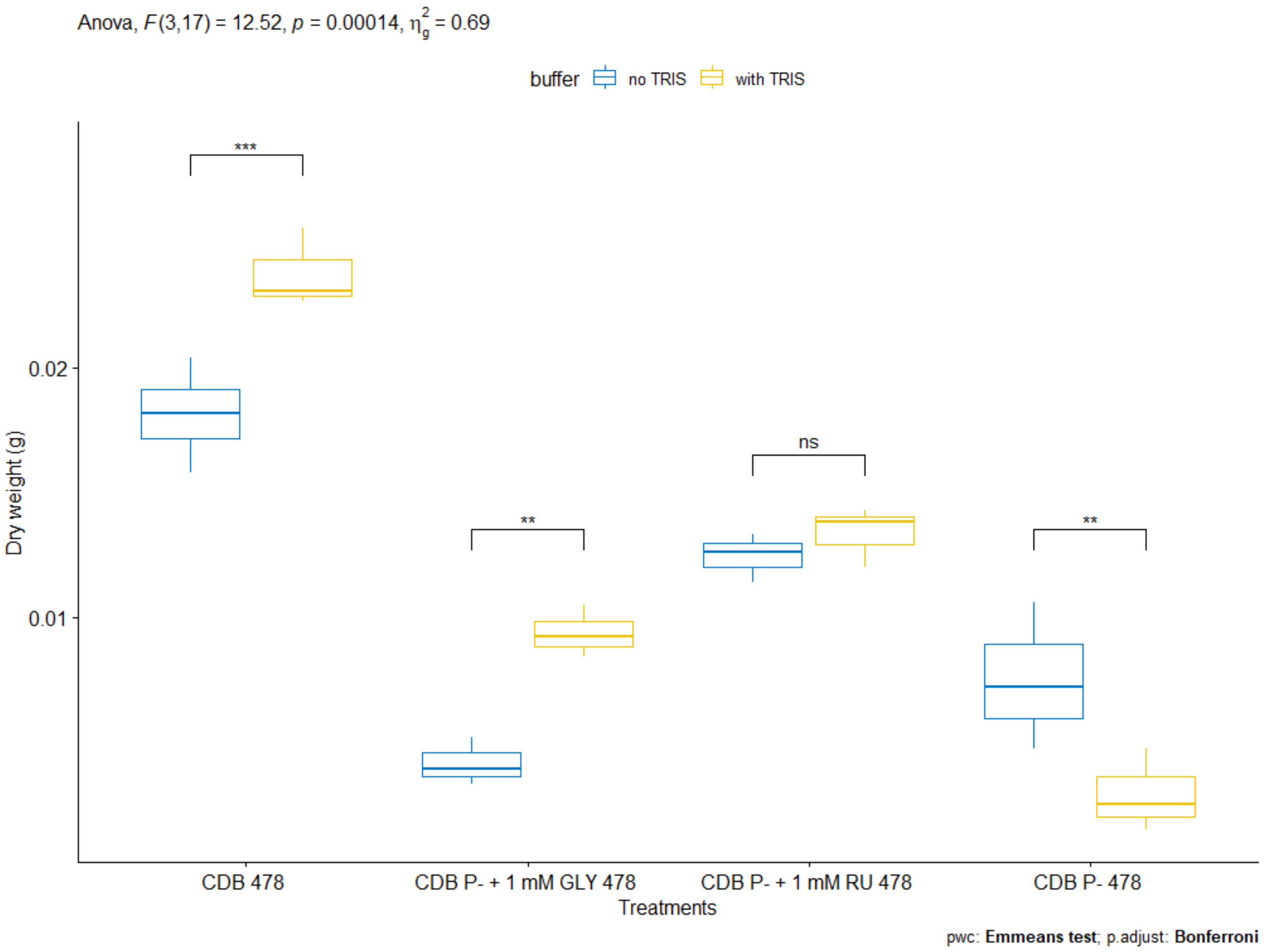

Figure S1.1: Boxplot of the Simple pairwise comparison output.
